# Intersectional Differences in Health Care Outcomes among Patients with Musculoskeletal Disorders in Germany

**DOI:** 10.3390/reports6020020

**Published:** 2023-04-29

**Authors:** Patrick Brzoska, Kübra Annac, Yüce Yilmaz-Aslan

**Affiliations:** 1 Health Services Research, Faculty of Health, School of Medicine, Witten/Herdecke University, 58455 Witten, Germany; 2 Department of Epidemiology and International Public Health, School of Public Health, Bielefeld University, 33615 Bielefeld, Germany; 3 Department of Nursing and Health Services Research, School of Public Health, Bielefeld University, 33615 Bielefeld, Germany

**Keywords:** migrants, intersectionality, Germany, trends, rehabilitation, arthropathies, dorsopathies, soft tissue disorders

## Abstract

In all regions of the world, musculoskeletal disorders are a significant contributor to the burden of chronic illnesses. The effectiveness of treatments, such as rehabilitation, may vary intersectionally across demographic and other categories. The present study examines this intersectionality with respect to a lack of improvement in health conditions after rehabilitation of patients in Germany. Routine data from 298,617 patients aged 18–65 years residing in Germany who received rehabilitation because of arthropathies, dorsopathies, or soft tissue disorders during 2006–2016 were included in the analysis. Odds of the outcome were compared across demographic groups and across diagnostic sub-groups by means of multivariable logistic regression. Interaction terms were included to examine intersectional differences across these groups and over time. Women were more likely than men to have an impairment despite treatment (adjusted odds ratio [aOR] = 1.11; 95%-CI = 1.08, 1.13). In addition, patients in semi-skilled/unskilled employment were at higher odds of a poor outcome compared to patients in skilled positions (aOR = 1.13; 95%-CI = 1.10–1.17). Nationality also affected health care outcomes, with Turkish nationals and nationals from a Yugoslav successor state having higher odds of a poor outcome than Germans (aOR = 1.56; 95%-CI = 1.45–1.67 and aOR = 1.52; 95%-CI = 1.41–1.65, respectively). The findings highlight the importance of an intersectional perspective in health research and practice and can support the development of strategies and measures that aim to reduce disparities in health care.

## 1. Introduction

Musculoskeletal disorders are becoming a more prominent factor in the increasing burden of chronic illnesses in all parts of the world. They lead to significant direct and indirect costs, as a result of lost productivity, disability retirement, and higher healthcare expenses associated with continuous medical treatment and rehabilitation. These conditions, which affect the muscles, bones, and joints, can range from common conditions like osteoarthritis to more rare and complex conditions such as systemic lupus erythematosus [1]. They often result in chronic pain, reduced mobility, and decreased quality of life, and can significantly affect an individual’s ability to work and participate in daily activities [2].

In Germany, annually, there are approximately 1.8 million hospital cases recorded for musculoskeletal conditions, which account for over 10% of the total direct healthcare costs [3]. Rehabilitation and other tertiary preventive services may contribute to preventing disability and early retirement related to musculoskeletal conditions and to promoting return to work [4]. However, the effectiveness of these services may differ based on various demographic factors, including sex, socioeconomic status, and immigration status, resulting in disparities in health care outcomes [5,6]. In addition, it is important to investigate potential intersectional variations across these groups given that determinants can also interact with each other (intersectionality) [7].

To better meet the needs of an increasingly heterogeneous population, providers of health care strive to offer services that better address the specific requirements of diverse patients [8,9]. Diversity-sensitive care can help to take into account the diversity of needs and expectations of patients by providing necessary conditions at the personnel and organizational level. It offers advantages in everyday care for patients by improving care and increasing health care satisfaction as well as for staff by reducing the workload through systemization and optimization processes [10].

It remains unclear how effective these initiatives are, and it is not yet clear whether they help to reduce existing disparities in healthcare outcomes. Building on our own previous work [11], the aim of the present study was to offer empirical evidence from Germany and to investigate the effectiveness of rehabilitation in patients diagnosed with arthropathies, dorsopathies, and soft tissue disorders, specifically examining outcomes across diverse demographic and socioeconomic categories. The results of this research can inform clinical practice and guide further investigations, potentially contributing to the development of targeted strategies aimed at addressing existing disparities in health care. Additionally, the findings may suggest potential opportunities to reduce costs for health care systems across Europe and beyond.

## 2. Materials and Methods

### 2.1. Data

For the analysis, a 10% random sample of data on rehabilitation among patients of working age (18–65 years) residing in Germany who underwent rehabilitation because of arthropathies (M00-M25), dorsopathies (M40-M54), or soft tissue disorders (M60-M79) between 2006 and 2016, was used. The data was provided by the German Federal Pension Insurance (Deutsche Rentenversicherung), which is responsible for insurance coverage of all employees who are required to pay social insurance contributions. As far as rehabilitation is concerned, the agency covers treatment (usually in the form of inpatient programs at specialized hospitals) for patients of working age (and cancer patients, irrespective of age), which makes up about two-thirds of all rehabilitations provided in the country annually [12,13].

### 2.2. Variables

The outcome of the study was the *improvement in health condition* (yes, no) after rehabilitation which was based on an evaluation at the time of discharge [14]. The evaluation was conducted by physicians as part of a medical discharge summary for each patient who completes rehabilitation and is recorded as one item.

We compared the outcome between *age* (in years), *sex* (male, female), *socioeconomic* (SES) and *nationality* groups (Germany, Turkey, a Yugoslav successor state, Portugal/Spain/Italy/Greece, other) by means of multivariable logistic regression (since we were only able to take nationality into account, immigrants who have acquired German citizenship, in the present study, are included in the nationality group “Germany”). As proxies for SES, the *type of employment* (full-time, part-time, unemployed, not applicable), the *type of occupation* (manual [e.g., carpenters and mechanics], services, technical/professional, administrative, other) and *occupational position* (skilled labor, semi-skilled/unskilled labor, trainee/unemployed) were considered. All analyses were also adjusted for *marital status*, *region of treatment* and *region of residence* (both referring to the 16 Federal states in Germany). In addition, as a proxy for health status the *time absent from work due to illness in the last 12 months* was included.

### 2.3. Analysis

To examine the relationship of the aforementioned variables and the outcome of rehabilitation descriptively, we used chi-square tests for categorical variables and an independent *t*-test for age. Logistic regression was used to examine factors associated with improvement or no improvement in health condition after rehabilitation by means of multivariable analysis. Adjusted odds ratios (aOR) with 95% confidence intervals (95%-CI) were used as effects estimates. Temporal trends in disparities and trends across demographic/socioeconomic groups as well as across the three diagnostic sub-groups were examined by means of interaction terms included in the model. We used average marginal effects (AME) to examine multiplicative interaction effects, given that unobserved heterogeneity may bias the evaluation of interaction terms based on odds ratios [15]. Analyses were conducted by means of Stata 16.

## 3. Results

Data on 298,617 cases were available for those who received treatment between 2006 and 2016. Of these, 50.8% were male. The mean age was 50.3 years. About 15% of patients experienced no improvement of their underlying condition after rehabilitation (Table 1). Women had higher odds of having an impairment despite treatment (adjusted odds ratio [aOR] = 1.11; 95%-CI = 1.08–1.13). In addition, patients in semi-skilled/unskilled employment had higher odds of having a poor outcome compared to those in skilled positions (aOR = 1.13; 95%-CI = 1.10–1.17). Nationality was significantly associated with the outcome of treatment, with Turkish nationals and nationals of a Yugoslav successor state having higher odds for a poor outcome than Germans (aOR = 1.56; 95%-CI = 1.45–1.67 and aOR = 1.52; 95%-CI = 1.41–1.65, respectively) (Table 2). The year patients underwent rehabilitation was not associated with disparities in treatment outcomes.

**Figure 1:**
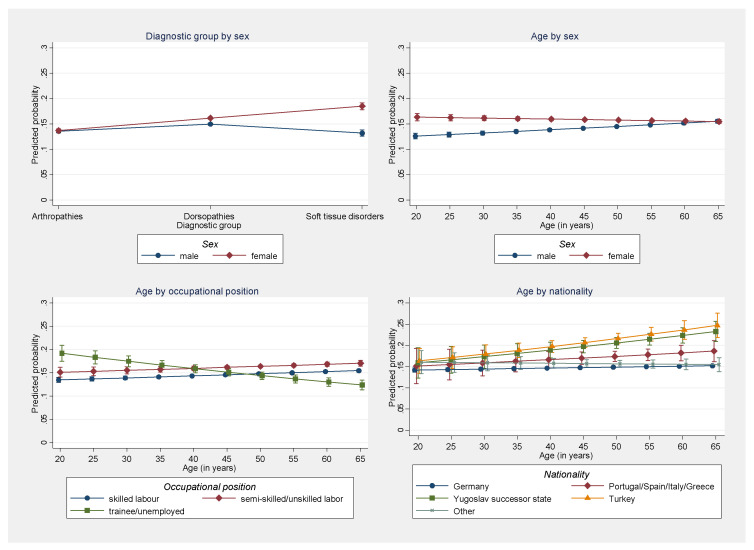
Predicted probability of a lack of improvement in health condition after rehabilitation by different interacting categories. Multivariable logistic regression model with two-way interaction terms (10% random sample of all individuals residing in Germany who underwent rehabilitation because of arthropathies, dorsopathies or soft tissue disorders during 2006–2016, n = 298,617). Note: For purposes of visualization, a small offset between the groups is used in the graphs to better illustrate overlapping confidence intervals.

**Figure 2:**
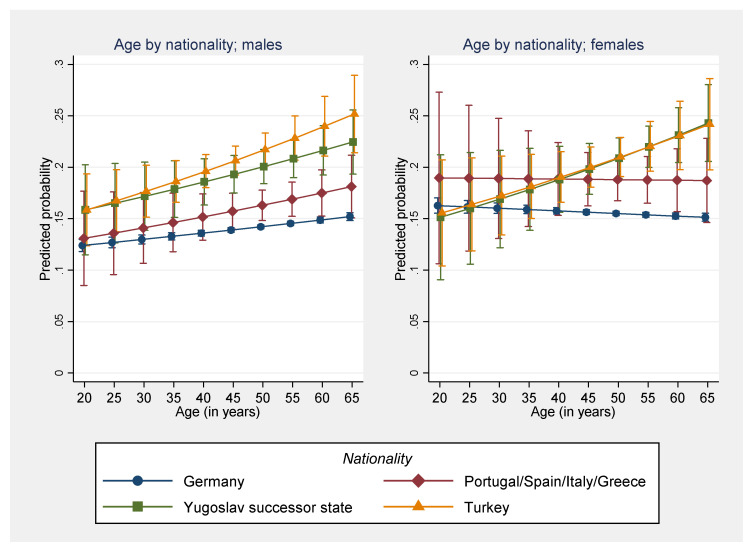
Predicted probability of a lack of improvement in health condition after rehabilitation by age, nationality and sex. Multivariable logistic regression model with a three-way interaction term (10% random sample of all individuals residing in Germany who underwent rehabilitation because of arthropathies, dorsopathies, or soft tissue disorders during 2006–2016, n = 298,617). Note: For purposes of visualization, a small offset between the groups is used in the graphs to better illustrate overlapping confidence intervals.

However, differences between males and females were larger for soft tissue disorders than for arthropathies and dorsopathies and decreased with age (Figure 1, top). Similarly, differences in treatment outcomes between patients who worked as trainees or were unemployed, in part, decreased with age, while differences between non-Germans and Germans increased with age (Figure 1, bottom). Conversely, these age-related interaction effects also mean that the effect of age varies between men and women, among occupational position groups, as well as between non-Germans and Germans. An exploratory analysis of multiplicative three-way interactions also revealed that for males differences between German nationals on the one hand and nationals from Turkey and a Yugoslav successor state exist for all age groups and tend to increase with age. For females in both groups, differences also increase with age; however, they only become pronounced after the age of 45 (Figure 2).

## 4. Discussion

Given the significant burden of musculoskeletal conditions, it is essential that individuals have access to effective tertiary preventive and management strategies. Using data from Germany, the present study shows that approximately 15% of all patients who underwent rehabilitation because of arthropathies, dorsopathies, or soft tissue disorders between 2006 and 2016 did not experience improvement in their health conditions after treatment. Poor rehabilitation outcomes may contribute to substantial direct and indirect costs [1]. The present investigation shows that the outcomes of such treatments may vary by different diversity characteristics. It indicates that female sex, a lower occupational position, a Turkish nationality or a nationality related to one of the Yugoslav successor states, and age are factors contributing to a higher chance of impairment despite treatment. Furthermore, the study shows that demographic and socioeconomic factors interact with each other and affect the outcomes intersectionally.

We found that women have a higher chance for poorer treatment outcomes compared to men. Disparities in rehabilitation outcomes by sex and gender have also been reported in previous research. Several studies also show that significant sex disparities exist in rehabilitation referrals. A meta-analysis showed that women were 32% less likely to be referred for cardiovascular treatment than men, despite equal rehabilitation eligibility [16]. Existing data suggest that women have lower adherence and completion rates in rehabilitation and are more likely to withdraw from rehabilitation because of their personal financial or occupational constraints [17]. Previous research also emphasized that psychosocial factors, such as social support and anxiety, also play a greater role in rehabilitation for women than for men [18]. Additionally, the unique gender-related challenges faced by women, such as balancing work and home responsibilities, may contribute to less favorable treatment outcomes [19].

Health disparities related to migration status, ethnicity, or race may also be present in the rehabilitative care setting [20,21,22,23]. In our study, such differences in treatment outcomes could also be observed, with Turkish nationals and nationals from a Yugoslav successor state having higher odds for a poor outcome than Germans. Our findings, in conjunction with previous research [24,25,26], suggest that immigrants face various obstacles within the healthcare system that may impede the delivery of adequate care. These barriers could stem from challenges in communication between patients and healthcare providers, limited knowledge of rehabilitation services, poor health literacy, and inadequate proficiency in the German language [27]. These barriers can hinder health care professionals’ ability to instruct patients on therapies and exercises, and to obtain crucial medical information, potentially diminishing the efficacy of rehabilitation. Aside from these challenges, communication may also be impaired by cultural needs and healthcare expectations that are not adequately addressed by health care professionals, such as illness perceptions related to cultural or religious beliefs [24,28].

Our study also shows that health care outcomes are associated with socioeconomic factors such as employment. This is in line with preceding studies [29,30] and may be explained by numerous challenges to health care and rehabilitation encountered by individuals with low socioeconomic status, such as poor health literacy [29,30,31].

When considering the broader context, our overall findings can be explained by the diversity of health care needs and preferences, thus illustrating the necessity for an intersectional perspective in health care [7]. An important finding from this analysis is that health care disparities are not caused by a single factor, but that many determinants are involved, which in part interact with each other. To address disparities in health care, therefore, the attention of health care facilities and professionals must be focused on multiple aspects. Consequently, only taking into account selected diversity characteristics such as migration status is not sufficient to provide adequate rehabilitative care to all population groups. In order to effectively address the needs of populations, including minorities, it is essential to consider their heterogeneity and to approach them at an individual and intersectional level—both in health research and health care practice [7,32]. What the present study and existing literature illustrate is the complexity of categories and diversity characteristics of patients, which is often insufficiently addressed. In particular, the mutually reinforcing effects of inequalities have not yet been sufficiently acknowledged, and the social determinants of health and characteristics of individual heterogeneous groups have not been adequately included—in either health research or health care practice. First, much of the existing literature, particularly in the field of health promotion and cultural competence, essentializes, for example, nationality or culture at the expense of other factors and barriers. Second, it simplifies different variables and their effects, often due to a lack of representative data that capture the complexity of experiences in minority groups or individuals in general [33].

By taking an intersectionality approach, stakeholders can better understand and address the needs and experiences of health care users, leading to more effective and equitable health care outcomes. In this way, a higher degree of patient centeredness in health care can be attained. Studies show that hospitals and rehabilitation facilities are well aware of the need for diversity-sensitive care. Yet, adequate strategies promoting diversity-sensitive care are rarely used [34,35]. This is largely due to a lack of practical hands-on overviews of suitable instruments that can be consulted for that purpose. A recent project addressed that limitation for rehabilitative care in Germany by developing a manual (‘DiversityKAT’) with tools and recommendations that can assist rehabilitation facilities in the implementation of diversity-sensitive health care approaches [36].

Designing diversity-sensitive health care strategies depends on the unique characteristics, structures, and objectives of health care facilities. These should be identified as part of a thorough needs assessment. Tools and techniques geared towards a diversity-sensitive approach often originate in the corporate world, but many can be adapted for use in the health care sector as well. Fundamental tools include mission statements that are sensitive to diversity, ethical guidelines, and policies that condemn discrimination and disadvantage for any particular population groups and that recognize diversity as a valuable opportunity for the organization. Concrete measures contributing towards diversity-sensitive care, for example, are adequate diversity trainings provided for health care staff. They aim to increase the awareness of staff about the diversity of their colleagues and patients by fostering respective values and attitudes, by promoting self-reflection, and by imparting appropriate skills, for example with respect to cross-cultural communication [10]. Similarly, mentoring programs can be implemented, where new employees are guided by experienced employees to help ease their transitions into the facility [37,38]. Furthermore, increasing diversity awareness in health care facilities can be accomplished through a personnel policy that is sensitive towards diversity, thereby avoiding diversity-related disadvantages in the hiring process and promoting diversity among staff, which again can have benefits in regard to catering to diverse patients. If employees are deployed according to their abilities, interests and available resources, they can better respond to needs and expectations of health care users, thus contributing to the optimization of health care processes. In designing adequate approaches, it is imperative to involve all relevant stakeholders, which do not only comprise health care facilities and insurance companies/health care providers, but also patients and their representing bodies.

The strengths of this study lie in the utilization of reliable and representative routine data, enabling both control for several confounding variables and analysis of trends over a period of ten years. However, it is essential to consider that as the only proxy variable for health status, only information on time of sickness-related absence from work in the year preceding rehabilitation was available. Consequently, residual confounding may exist due to variations in health status before rehabilitation, which could impact disparities in health outcomes among different population groups. Additional information related to rehabilitation, e.g., patients’ initial health status, their attitudes towards treatment, as well as information about the treatment itself (type and number of therapies, etc.), could provide further insights into intersectional differences in health outcomes. Unfortunately, this information was not available in the present study. Another limitation concerns information on migration status. At present, the only feasible way to identify immigrants in routine data from the German Federal Pension Insurance is by their nationality. As a result, immigrants who have acquired German citizenship and who make up more than half of the immigrant population in Germany [39], in the present study were included in the nationality group “Germany”.

## 5. Conclusions

Musculoskeletal conditions are becoming a larger contributor to the global burden of chronic diseases, causing significant financial impacts in both direct and indirect ways, including lost productivity, early retirement due to disability, and increased healthcare expenses from ongoing care. Effective rehabilitation services make a significant contribution to enhancing patients’ mobility, quality of life, and ability to work, and empowering them in their health maintenance. Outcomes of health care, including rehabilitation, may vary intersectionally across demographic and socioeconomic groups. Previous research suggests that, for example, immigrants face various obstacles within the healthcare system that may impede the delivery of adequate care. The present investigation delivers empirical evidence from Germany and examines outcomes of rehabilitation of arthropathies, dorsopathies, and soft tissue disorders in patients across these categories. By means of nationwide and representative data, it identified several disparities between demographic/socioeconomic groups in health care outcomes over time, some of which also intersectionally overlap with each other. Although some limitations need to be considered, the study shows that differences in health care outcomes do not vary over time, indicating that current approaches implemented by health care providers are not successful in reducing disparities. Formulating effective methods to eliminate disparities in healthcare can also lower expenses for the healthcare system. Future efforts, therefore, must rely on approaches which are not only holistic, systematic, and sensitive to the diversity of health care users, but for which appropriate evaluation studies have been conducted. For this purpose, it is necessary to facilitate the translation of research findings into practice.

## Figures and Tables

**Table 1:** **Sample description by treatment outcome** (10% random sample of all individuals residing in Germany who underwent rehabilitation because of arthropathies [ICD-10 M00-M25], dorsopathies [ICD-10 M40-M54], or soft tissue disorders [ICD-10 M60-M79] during 2006–2016, n = 298,617).

	Improvement in Health Condition after Rehabilitation (n, %)		Total	*p*-Value
	Yes	No		
**Sex** (n, %)				<0.01
Male	129,345 (51.0%)	22,392 (49.7%)	151,737 (50.8%)	
Female	124,213 (49.0%)	22,667 (50.3%)	146,880 (49.2%)	
**Age** (mean, sd)	50.3 (8.8)	50.2 (8.8)	50.3 (8.8)	<0.01
**Nationality** (n, %)				<0.01
Germany	238,324 (94.0%)	41,348 (91.8%)	279,672 (93.7%)	
Portugal/Spain/Italy/Greece	2931 (1.2%)	649 (1.4%)	3580 (1.2%)	
Yugoslav successor state	3022 (1.2%)	826 (1.8%)	3848 (1.3%)	
Turkey	3369 (1.3%)	1080 (2.4%)	4449 (1.5%)	
Other	5912 (2.3%)	1156 (2.6%)	7068 (2.4%)	
**Occupational position** (n, %)				<0.01
Skilled labor	191,032 (75.3%)	32,128 (71.3%)	223,160 (74.7%)	
Semi-skilled/unskilled labor	46,228 (18.2%)	10,179 (22.6%)	56,407 (18.9%)	
Trainee/unemployed	16,298 (6.4%)	2752 (6.1%)	19,050 (6.4%)	
**Type of employment** (n, %)				<0.01
Fulltime	174,270 (68.7%)	29,691 (65.9%)	203,961 (68.3%)	
Part-time	43,267 (17.1%)	7883 (17.5%)	51,150 (17.1%)	
Unemployed	16,046 (6.3%)	4201 (9.3%)	20,247 (6.8%)	
Other	19,975 (7.9%)	3284 (7.3%)	23,259 (7.8%)	
**Occupation** (n, %)				<0.01
Manual	76,993 (30.4%)	14,299 (31.7%)	91,292 (30.6%)	
Services	59,649 (23.5%)	11,437 (25.4%)	71,086 (23.8%)	
Technical/professional	38,746 (15.3%)	6495 (14.4%)	45,241 (15.2%)	
Administrative	40,963 (16.2%)	6922 (15.4%)	47,885 (16.0%)	
Other	37,207 (14.7%)	5906 (13.1%)	43,113 (14.4%)	
**Diagnosis** (n, %)				<0.01
Arthropathies	62,033 (24.5%)	9823 (21.8%)	71,856 (24.1%)	
Dorsopathies	173,488 (68.4%)	31,354 (69.6%)	204,842 (68.6%)	
Soft tissue disorders	18,037 (7.1%)	3882 (8.6%)	21,919 (7.3%)	
**Marital status** (n, %)				<0.01
Not married	74,411 (29.3%)	14,157 (31.4%)	88,568 (29.7%)	
Married	175,055 (69.0%)	30,183 (67.0%)	205,238 (68.7%)	
Other	4092 (1.6%)	719 (1.6%)	4811 (1.6%)	
**Time absent from work in the last 12 months** (n, %)				<0.01
None	37,724 (14.9%)	5675 (12.6%)	43,399 (14.5%)	
<3 months	141,324 (55.7%)	17,990 (39.9%)	159,314 (53.4%)	
3–6 months	37,581 (14.8%)	8704 (19.3%)	46,285 (15.5%)	
6+ months	28,893 (11.4%)	11,460 (25.4%)	40,353 (13.5%)	
Not employed	8036 (3.2%)	1230 (2.7%)	9266 (3.1%)	

Note. sd: standard deviation.

**Table 2:** Results of the multivariable logistic regression models with no improvement in health condition after rehabilitation as the dependent variable. Adjusted odds ratios (aOR), adjusted average marginal effects (aAMR), and their respective 95% confidence intervals [95%-CI] (10% random sample of all individuals residing in Germany who underwent rehabilitation because of arthropathies [ICD-10 M00-M25], dorsopathies [ICD-10 M40-M54] or soft tissue disorders [ICD-10 M60-M79] during 2006–2016, n = 298,617).

Independent Variable ^1^	aOR ^2^	95%-CI		aAME ^2^	95%-CI	
**Sex (Ref.: Male)**						
Female	1.11	1.08	1.13	0.012	0.009	0.016
**Age (in years)**	1.002	1.001	1.003	0.0003	0.0001	0.0004
**Nationality (Ref.: Germany)**						
Portugal/Spain/Italy/Greece	1.22	1.12	1.33	0.026	0.014	0.038
Yugoslav successor state	1.53	1.41	1.65	0.059	0.046	0.072
Turkey	1.56	1.45	1.68	0.062	0.051	0.074
Other	1.07	1.00	1.14	0.008	0.000	0.017
**Occupational position (Ref: Skilled labor)**						
Semi-skilled/unskilled labor	1.13	1.10	1.17	0.016	0.013	0.020
Trainee/unemployed	1.03	0.96	1.10	0.003	−0.005	0.012
**Type of employment (Ref.: Full time)**						
Part-time	1.04	1.01	1.08	0.005	0.001	0.009
Unemployed	1.30	1.25	1.35	0.034	0.029	0.040
Other	1.01	0.95	1.07	0.001	−0.006	0.008
**Occupation (Ref.: Manufacturing)**						
Services	1.00	0.97	1.03	0.000	−0.004	0.003
Technical/professional	0.96	0.93	1.00	−0.004	−0.009	0.000
Administrative	0.96	0.93	1.00	−0.005	−0.009	0.000
Other	0.92	0.89	0.96	−0.010	−0.014	−0.006
**Diagnosis (Ref: Skeletal system)**						
Dorsopathies	1.17	1.14	1.20	0.019	0.016	0.022
Soft tissue disorders	1.20	1.15	1.25	0.022	0.017	0.027
**Significant interaction effects (*p* < 0.05)**						
*Sex by diagnostic group*						
Arthropathies, female vs. male				0.0000	−0.0042	0.0061
Dorsopathies, female vs. male				0.0110	0.0084	0.0155
Soft tissue, female vs. male				0.0530	0.0438	0.0623
*Age by sex*						
Male, age				0.0010	0.0005	0.0008
Female, age				−0.0002	−0.0004	0.00003
*Age by occupational group*						
Skilled labor, age				0.0005	0.0003	0.0006
Semi-skilled/unskilled labor, age				0.0004	0.0001	0.0008
Trainee/unemployed, age				−0.0014	−0.0019	−0.0010
*Age by nationality*						
Germany, age				0.0002	0.0001	0.0004
Portugal/Spain/Italy/Greece, age				0.0008	−0.0006	0.0022
Yugoslav successor state, age				0.0017	0.0004	0.0030
Turkey, age				0.0020	0.0006	0.0033
Other, age				−0.0001	−0.0010	0.0008

Note. ^1^ Main effects have been calculated based on a main effects model with no interaction effects included. Interaction effects have been calculated based on a main effects model with interaction effects. ^2^ Odds ratio (aOR) and average marginal effects (aAME) are adjusted for the variables displayed in the tables as well as for marital status, region of residence, region of treatment and the time absent from work due to illness in the last 12 months.

## Data Availability

Researchers who meet the eligibility requirements for access to confidential data may obtain the data supporting the findings of this study free of charge from the German Federal Pension Insurance. More information can be found at https://www.eservice-drv.de/FdzPortalWeb/dispcontent.do?id=main_fdz_english (accessed on 26 April 2023).
